# Potential Contribution of IL-27 and IL-23 Gene Polymorphisms to Multiple Sclerosis Susceptibility: An Association Analysis at Genotype and Haplotype Level

**DOI:** 10.3390/jcm11010037

**Published:** 2021-12-22

**Authors:** Ioana S. Barac, Mihaela Iancu, Vitalie Văcăraș, Angela Cozma, Vasile Negrean, Dorel Sâmpelean, Dafin F. Mureșanu, Lucia M. Procopciuc

**Affiliations:** 1 Department of Clinical Neurosciences, “Iuliu Hațieganu” University of Medicine and Pharmacy Cluj-Napoca, 400000 Cluj-Napoca, Romania; siminabarac@gmail.com (I.S.B.); dafinm@ssnn.ro (D.F.M.); 2 Department of Medical Informatics and Biostatistics, Faculty of Medicine, “Iuliu Hațieganu” University of Medicine and Pharmacy Cluj-Napoca, 400012 Cluj-Napoca, Romania; 3 Department of Internal Medicine, “Iuliu Hațieganu” University of Medicine and Pharmacy Cluj-Napoca, 400000 Cluj-Napoca, Romania; angelacozma@yahoo.com (A.C.); vasile.negrean@umfcluj.ro (V.N.); dorel.sampelean@gmail.com (D.S.); 4 Department of Biochemistry, “Iuliu Hațieganu” University of Medicine and Pharmacy Cluj-Napoca, 400000 Cluj-Napoca, Romania; luciamariaprocopciuc@yahoo.com

**Keywords:** Il-23R, IL-27, rs181206, rs153109, rs11209026, multiple sclerosis, prognosis

## Abstract

(1) Background: interleukin 23 (IL-23) and interleukin 27 (IL-27) modulate the activity of T helper 17 cells (Th17) with critical roles in autoimmune diseases and multiple sclerosis (MS). The genes responsible for cytokine generation are highly influenced by the presence of single nucleotide polymorphisms (SNP) in main regions such as regulatory sequences or in promoter regions, contributing to disease susceptibility and evolution. The present study analyzed the associations of IL-23 and IL-27 SNPs with susceptibility to multiple sclerosis. (2) Methods: We performed a case-control study including 252 subjects: 157 patients diagnosed with MS and 95 controls. We used polymerase chain reaction-restriction fragment length polymorphism (PCR-RFLP) to determine the genotypes for IL-27 *T4730C* (rs 181206), IL-27 *A964G* (rs 153109), and IL-23 receptor gene (IL-23R) *G1142A* (rs 11209026). (3) Results: The IL27-*T4730C* gene polymorphism was significantly associated with an increased odds of MS under the dominant genetic model (TC + CC variant genotypes, adjusted odds ratio OR = 4.06, 95% CI: 2.14–7.83, *p*-value = 0.000007, Q-value = 0.000063). Individuals carrying the IL-27 *A924G* variant (AG + GG) genotype presented higher odds of MS compared to non-carriers under the dominant model (adjusted OR = 1.93, 95% CI: 1.05–3.51, *p*-value = 0.0324, Q-value = 0.05832) and the allelic genetic model (unadjusted *p*-value = 0.015, OR = 1.58, 95% CI: 1.09–2.28), while IL-23-*R381Q* SNP conferred a decreased odds of MS under a codominant model of inheritance (adjusted OR = 0.26, 95% CI: 0.08–0.92, *p*-value = 0.0276, Q-value = 0.058) and an allelic model (unadjusted *p*-value = 0.008, OR = 0.23, 95% CI: 0.07–0.75). In an additive model with adjustment for age group (≤40 years vs. >40 years), sex and smoking, patients carrying the G-C (*A964G*, *T4730C*) haplotype had a 3.18 increased risk (95% CI: 1.74–5.81, *p* < 0.001) to develop multiple sclerosis. (4) Conclusions: The results of the current study showed a significant relationship of IL-27-*A964G* and IL-27-*T4730C* polymorphisms with increased risk of MS, and also the protective role of the IL-23-*R381Q* polymorphism. Moreover, the haplotype-based analysis proposed the mutant G-C (*A924G*, *T4730C*) as a significant risk haplotype for the development of MS.

## 1. Introduction

Multiple sclerosis (MS) is the most commonly acquired demyelinating disorder of the central nervous system (CNS) in the young adult [1].

The prevalence of MS in Western Europe is estimated at 0.1–0.2% of the population (100–200 cases per 100,000 people), leading to disability and socioeconomic burden [2]. Due to the increasing awareness of MS in the society and also due to the available diagnostic tools, the reported prevalence is significantly increasing.

The etiology of MS is determined by a complex interplay between environmental factors, genetic factors and lifestyle, leading to demyelinating lesions, axonal loss, astrocytic gliosis and microglial activation [3,4]. The environmental factors leading to immune dysregulation are represented by: viral infections with Epstein Barr virus (EBV), herpes simplex virus (HSV) types 1, 2 and 6, varicella zoster virus (VZV), lack of sun exposure and vitamin D deficiency [5,6,7]. The environmental factors and lifestyle factors (diet, smoking and obesity) maintain a pro-inflammatory status characterized by high levels of circulating cytokines [4,8]. The persistent inflammation determines the loss of trophic support provided by myelin, impairing axonal survival and contributing to a failure of remyelination [9].

The involvement of genetic factors in MS is supported by studies evaluating familial clustering, which showed a 10 to 15 times higher risk for the siblings of MS patients to develop MS, compared to the general population [10]. Evaluating the genetic risk factors, genome wide association studies (GWAS) revealed numerous susceptibility loci associated with MS, many of these represented by single nucleotide polymorphisms (SNP), showing that most of these genetic variations are related to adaptive immunity genes [11,12]. Another argument for the role played by the adaptive immune system in the pathogenesis of MS is the successful therapeutic response of strategies targeting T and B cells [4]. Altogether, GWAS support the idea that susceptibility to MS is influenced by the action of common allelic variants in multiple genes defined by their size effect, known as multiple sclerosis genetic burden, which can be calculated for each individual [13,14].

Delineating a genetic profile of the patients at risk to develop MS has profound implications, helping in narrowing the time window until diagnosis and treatment.

A few previous studies revealed that genes encoding cytokines which modulate the activity of T-helper 17 (Th17) cells may influence MS predisposition, enhancing the destruction or repair of myelin [15]. The IL-12 family of cytokines, comprising IL-12, IL-23, IL-27 and IL-35, are small proteins, bridging the innate and adaptive immunity and promoting immune responses [16].

IL-23 is a covalent heterodimer of IL-12p40 and IL-23p19, produced mainly by antigen presenting cells (APC) [17]. IL-23 attached to IL-23 receptor (IL-23R) stimulates the activation of Janus Kinase signal transducer 2 (JAK2) and the phosphorylation of IL-23R at tyrosine 705 (Tyr705). This attachment leads to the recruitment and phosphorylation of the activator factors of transcription 3 (STAT 3) and 4 (STAT 4) which translocate to the nucleus. In the nucleus, STAT 3 and STAT4 stimulate the transcription of pro-inflammatory cytokine genes, including IL-17 [18].

In experimental autoimmune encephalomyelitis (EAE), the animal model for MS, IL-23 is essential in promoting Th17 cell population expansion [19]. Th17 activated cells, stimulate the cytokines and chemokines production, determining blood brain barrier (BBB) dysfunction and enhancing the influx of CD4+ lymphocytes, neutrophils and activated monocytes in the CNS [20].

*G1142A* (rs11209026, *R381Q*) encoding a subunit of IL-23R, is located in exon 9 of IL-23R. Close to the end of the coding sequence of exon 9, the presence of *A allele* instead of the more common *G allele* leads to the amino acid modification of arginine at residue 381 into glutamine (*R381Q*) in the protein product. This amino acid modification has functional consequences [21]. Therefore, the substitution of arginine (Arg) with glutamine (Gln) modifies the signaling and responses to IL-23 [22,23]. The substitution of arginine (Arg) with glutamine (Gln) is located in the cytoplasmic part of IL-23R protein, close to the first tyrosine phosphorylation site, between JAK2 binding site and the transmembrane domain [21,24]. This unique disposition influences either the signal transduction or the surface localization of IL-23R, with functional consequences [24]. Therefore, the substitution of arginine (Arg) with glutamine (Gln) modifies the signaling and responses to IL-23 [22,23].

In MS, IL-23R was studied for its involvement in the process of CNS and optic nerve inflammation, stimulating the homing and survival of myelin specific T lymphocytes in the CNS [25,26,27].

IL-27 is another member of the IL-12 family, known for its pro- and anti-inflammatory actions, with contrasting roles in modulating the activity of T cells.

IL-27 activates multiple signaling cascades, such as tyrosine kinase-2, JAK1 and JAK2 [28,29]. IL-27 is involved in the regulation of T helper cells, dendritic cells and macrophages through STAT1 and STAT3 pathways [28]. IL-27 suppresses T-helper 2 (Th2) production and Th17 differentiation and stimulates T-helper 1 (Th1) cell differentiation through STAT1 pathway [30]. In contrast, STAT3 pathway activation plays an important role in Th17 cell differentiation, promoting and increasing IL-17 gene transcription [31]. In order to maintain an appropriate immune response, the activation of STAT3 works as a counterbalance to activation of STAT1 pathway [28].

In the CNS, IL-27 has neuroprotective effects, stimulating the production of neurotrophic factor and the production of nerve growth factor, enhancing remyelination [32,33].

The IL-27 gene is located on chromosome 16p11 and consists of five exons [34]. This gene has several single nucleotide polymorphisms (SNPs).

*A964G* (rs153109) is a functional polymorphism, located 964 bp upstream to the transcription site of the IL-27 gene, consisting of the transition of *A* to *G.* This transition leads to the formation of a new binding site in the IL-27 gene promoter, consequently changing the IL-27 gene expression pattern [35,36]. The localization of this SNP in the promoter region of the IL-27 gene is near important epigenetic marks, such as H3K4me3 and H3K27ac, influencing the transcriptional activity of the promoter and enhancer [37,38], controlling the protein expression and transcription, which is why it was evaluated in different types of cancers [39].

*T4730C* (rs181206, Leu119Pro), located in exon 4, is a missense polymorphism, consisting of a single nucleotide alteration from *T to C* at nucleotide 4730 [40,41,42]. As a consequence of nucleotide substitution, the amino acid leucine is changed to proline, in position 119 (Leu119Pro) [40,41].

IL-27-*A964G* and IL-27-*T4730C* were associated with digestive and gynecological neoplasms and with other autoimmune diseases such as Behcet disease (BD), ulcerative colitis (UC) and rheumatoid arthritis (RA) [36,43,44]. To date, to our knowledge, there are no studies investigating the relationships between IL-27-*T4730C*, IL-27-*A964G*, IL-23-*R381Q* gene polymorphisms and the risk of multiple sclerosis. Accordingly, the present study analyzed: (i) the potential association of these gene polymorphisms to multiple sclerosis susceptibility and (ii) the relationships of IL-27 and IL-23R SNPs with the clinical features of patients with multiple sclerosis.

## 2. Materials and Methods

One hundred and fifty-two patients with multiple sclerosis (MS), 106 (67.5%) females and 51 (32.5%) males (mean age, 42 years), and ninety-five controls, 61 (64.2%) females and 34 (35.8%) males (mean age, 27 years), were included in our study between March 2019 and April 2021. We recruited the patients from the National Program of Multiple Sclerosis in the Cluj-Napoca Neurology Clinic, Romania. MS diagnosis was based on clinical and radiological findings, according to the 2017 Mc Donald criteria [45]. The information was obtained from neurological examinations and personal interviews. Data about radiological investigations were obtained from patients’ files. The inclusion criteria were patients with the diagnosis of MS, without any previous treatment or with one of the following treatments for MS: interferon, glatiramer acetate. The exclusion criteria were other autoimmune diseases such as systemic lupus erythematosus (SLE), RA, ankylosing spondylitis (AS), inflammatory bowel disease (IBD), psoriasis, type 1 diabetes mellitus, clinical relapse at the time of evaluation, use of cortisone in the last month, other immunosuppressive therapies recently used.

The control group was composed of healthy volunteers without a personal or family history of multiple sclerosis. Both groups shared a common geographical area and had the same ethnicity and socioeconomic status.

Our study was approved and registered in 25 February 2019 by the ethical committee, with the registration number 31.

### 2.1. Methods

#### 2.1.1. DNA Extraction

In order to identify the genotypes for the three genetic variations, IL-27-*T4730C*, IL-27-*A964G* and IL-23-*R381Q*, 2 mL of peripheral blood were collected through venipuncture in ethylenediamine tetraacetic acid (EDTA) anticoagulated tubes for both patients with multiple sclerosis and controls. High molecular weight DNA was isolated using a Zymoresearch kit (Quick-DNAMiniprep, Kit-Zymo Research Corporation, Freiburg, Germany). The probes were stored at −20 °C until PCR analysis was done.

#### 2.1.2. Polymerase Chain Reaction-Restriction Fragment Length Polymorphism (PCR-RFLP Analysis)

The IL-27-*T4730C*, IL-27-*A964G* and IL-23-*R381Q* polymorphisms were identified using polymerase chain reaction (PCR) and restriction fragment length polymorphism analysis (RFLP), according to the methods described by Mohamadi et al., Anber et al. and Mosayebian et al. [46,47,48].

The PCR reaction was carried out in 25 μL mixture containing 20 ng genomic DNA, 200μM dNTPs (dATP, dGTP, dCTP, dTTP), 2.0 mM MgCl_2_, 0.2 μM primers, 0.65U Taq DNA polymerase (Taq buffer, 20 mM Tris-HCl (pH 8.0), 1 mM DTT, 0.1 mM EDTA, 100 mM KCl, 0.5% (*v*/*v*) Nonidet P40, 0.5% (*v*/*v*) Tween20 and 50% (*v*/*v*) glycerol). Fast PCR amplification was performed in an iCycler C1000 BioRad (Bio-Rad Life Science, Hercules, CA, USA). The specificity of the amplification reaction was checked by electrophoresis of 10 µL PCR product on 2% agarose gel stained with 10 mg/mL ethidium bromide solution and the gel was visualized on a UV light source.

The PCR products were incubated in 10 μL volume for 3 h with 5U restriction endonucleases specific for each genetic variation.

**Figure 1:**
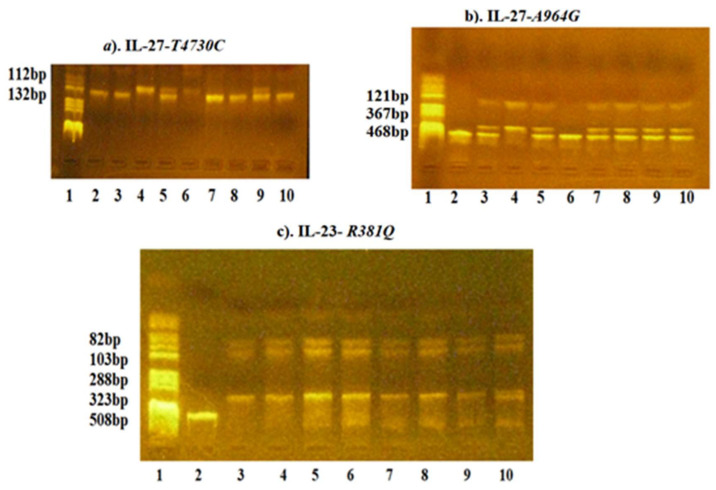
Agarose gel electrophoresis for identification of the genotypes for IL-27-*T4730C*, IL-27-*A964G* and IL-23-*R381Q* polymorphisms. (**a**). IL-27-*T4730C*: Lane 1—pBRHaeIII Digest DNA molecular marker V; Lane 2, 3, 7, 8, 10—TT genotype: fragment of 132bp; Lane 4, 6—CC genotype: fragment of 112bp; Lane 5, 9—TC genotype: fragments of 132 and 112bp; (**b**). IL-27-*A964G*: Lane 1—pBRHaeIII Digest DNA molecular marker V; Lane 2, 6—AA genotype: fragment of 468 bp; Lane 3, 5, 7, 8, 9, 10—AG genotype: fragments of 468 bp, 367 bp, 121 bp; Lane 4—GG genotype: fragments of 367 bp and 121bp; (**c**). IL-23-*R381Q*: Lane 1—pBRHaeIII Digest DNA molecular marker V; Lane 2—PCR: fragment of 508 bp; Lane 3-9—GG genotype: fragments of 288 bp, 103 bp, 82 bp; Lane 10—AG genotype: fragments of 323 bp, 288 bp, 103 bp, 82 bp.

The sequences of the primers, the PCR protocols and the RFLP conditions are presented in Table 1. The results are also presented in Figure 1.

The restriction enzymes were supplied by New England Biolabs (New England Biolabs UK, Ltd., Hitchin, UK) and the primers were from Eurogentec (Kaneka Eurogentec S.A. Biologics Division, Liege, Belgium).

### 2.2. Statistical Analysis

Quantitative continuous variables that followed a Gaussian distribution were summarized by arithmetic mean and standard deviation (SD). For the quantitative continuous variables with deviations from Gaussian distribution, we used the median value and interquartile interval IQR = [Q1; Q3] (Q1 = 25th percentile, Q3 = 75th percentile) as centrality and dispersion measures. Qualitative nominal variables were presented using relative (%) and absolute frequencies (number of cases).

The comparisons of the distributions of demographic, lifestyle and clinical variables in relation to the MS or variant genotypes of the investigated SNPs were performed using Student’s *t*-test for independent samples, Mann–Whitney U test, Chi-square or Fisher’s exact tests.

The departure from Hardy–Weinberg equilibrium (HWE) and the linkage disequilibrium (LD) were assessed for the investigated SNPs using the Chi-square test from “genetics” R package.

The association between a particular gene’s variants and odds of MS was evaluated by unconditional binomial logistic regression and the effect size of association was presented using the odds ratio (OR) and 95% confidence interval (CI) without/with adjustment for age group (≤40 years vs. >40 years), sex and smoking. Logistic regression analysis was performed under four different inheritance genetic models (codominant, dominant, recessive and overdominant) using the R-project package “SNPassoc” version 2.0.2. The same package was used to evaluate the potential pairwise interaction between the investigated gene variants.

For all two-sided statistical tests, the significance level (α) was set at 0.05. In order to account for multiple testing, the *p*-values were adjusted using the false discovery rate (FDR) method and an estimated Q-value was calculated for each SNP using R-project package “qvalue” version 2.15.0. Significance of the associations was achieved for a Q-value threshold of 0.10, as previously suggested [49].

Haplotype analysis was performed in order to estimate haplotype frequencies using the expectation-maximization algorithm of R-project package “haplo.stats” version 1.8.6. The most common haplotype with a significant negative association with the disease risk served as the “reference” haplotype. The association of haplotypes with odds of MS was tested using an additive generalized linear model (GLM) implemented in the R-project package “haplo.stats”.

All statistical analyses were performed in R software, version 4.1.1 (R Core Team (2021). R: A language and environment for statistical computing. R Foundation for Statistical Computing, Vienna, Austria. URL https://www.R-project.org/ (accessed on 18 August 2021)).

## 3. Results

### 3.1. Study Subjects

The demographic, clinical and lifestyle characteristics of the study subjects (*n* = 252) are summarized in Table 2. The control group (*n_1_* = 95) and the group of patients with multiple sclerosis (MS) (*n_2_* = 157) differed significantly in age distribution, residence area, alcohol consumption (*p*-value < 0.001) and smoking frequencies (*p*-value = 0.040).

Regarding disease duration and evolution, 62 (39.49%) MS patients were in the first 5 years from onset, 62 (37.1%) were free of relapses with a stable evolution, but on follow-up MRI (magnetic resonance imaging), 29 (17.4%) patients had enlarged lesions. On MRI, the distribution of demyelinating lesions in 55 (35%) patients was supratentorial, infratentorial and spinal. At the neurological evaluation, 97 (61.8%) patients had motor weakness, 61 (38.9%) had cerebellar syndrome, 95 (60.5%) had sensory symptoms, 35 (22.3%) had depression, nine (17.6%) had memory loss, and ten (19.6%) had urge incontinence. For 54 (34.4%) patients, the onset of the disease was with optic neuritis. Among the risk factors encountered in our study, 52 (31.1%) MS patients were smokers and 35 (21%) consumed alcohol occasionally.

### 3.2. Association Analysis between the Studied SNPs and Odds of MS

The distributions of genotypes for IL-27-*T4730C*, IL-27-*A964G* and IL-23-*R381Q* SNPs in the control group showed a significant departure from the assumptions of the Hardy–Weinberg equilibrium (HWE) for IL-23-*R381Q* (no individuals carrying the homozygous GG genotype were identified among controls) and for IL-27-*T4730C* SNP (*p* = 0.0264). For IL-27-*A964G*, the genotypic frequency of control subjects did not show significant deviations from HWE (*p* = 0.831).

In particular, the increased odds of MS were significantly associated with carriage of TC + CC variant genotypes (adjusted OR = 4.09, 95% CI: 2.14–7.83, *p*-value = 0.000007, Q-value = 0.000063). In addition, allele C of the IL-27-*T4730C* gene polymorphism showed a significant association with the odds of MS (unadjusted *p* < 0.001, OR = 2.92, 95% CI: 1.77–4.81). Individuals carrying the IL-27-*A964G* variant (AG + GG) genotype presented higher odds of MS compared to non-carriers under the dominant model (adjusted OR = 1.93, 95% CI: 1.05–3.51, *p*-value = 0.0324, Q-value = 0.05832) and the allelic genetic model (unadjusted *p*-value = 0.015, OR = 1.58, 95% CI: 1.09–2.28), while IL-23-*R381Q* SNP conferred a decreased odds of MS under a codominant model of inheritance (adjusted OR = 0.26, 95% CI: 0.08–0.92, *p*-value = 0.0276, Q-value = 0.058) and an allelic model (unadjusted *p*-value = 0.008, OR = 0.23, 95% CI: 0.07–0.75). The distribution of the genotypes for the three polymorphisms is presented in Table 3.

### 3.3. SNP-SNP Interactions between IL-27-T4730, IL-27-A964G and IL-23-R381 and MS Risk

We then tested all possible pairwise interactions between IL-27-*T4730*, IL-27-*A964G* and IL-23-*R381Q* gene polymorphisms in dominant, codominant, and recessive models. No significant pairwise epistatic interactions were found under the dominant, codominant and recessive models (Table 4).

### 3.4. Analysis of Haplotypes and Linkage Disequilibrium (LD) between IL-27-T4730, IL-27-A964G SNPs

We performed pairwise LD analysis for IL-27-*T4730C*, IL-27-*A964G* SNPs and we found a significant non-random association of the studied alleles (D’ = 0.64, r = 0.383, *p* < 0.05). Haplotype analysis of the two gene polymorphisms revealed four haplotypes, which were present in control and MS patients (Table 5).

### 3.5. Association Analysis of MS Characteristics with SNPs of IL-27-T4730, IL-27-A964G and IL-23-R381Q

The associations of the investigated SNPs with demographic and clinical characteristics of MS patients are summarized in Table 6.

We found no significant association between the studied SNPs and clinical characteristics in MS patients, the distributions of variant genotypes under the dominant and codominant genetic models being similar in patients with different durations of MS or forms of disease (*p* > 0.05). In the recessive genetic model, we found a marginal significance only for the association between smoking and IL-27-*T4730C* (Chi-square test, *p*-value = 0.054), the frequency of the variant CC genotype being higher in smokers (52.6%) than in non-smokers (30.4 %). Under the overdominant genetic model, there was a significant association between the form of MS and the IL-27-*A964G* polymorphism (*p* = 0.0301), the frequency of the variant AG genotype tending to be higher in MS patients with the RR form compared to patients with the SP form (Chi-square test, adjusted *p*-value = 0.0516, 57.8% vs. 27.3%).

## 4. Discussion

The pathogenic mechanisms in multiple sclerosis (MS) are progressive, evolving from tissue damage and peripheral antigen release to priming new immune responses in the lymphoid tissue and lymphocyte invasion into the central nervous system (CNS) [4,50].

Human and murine model studies emphasized the role of Th17 cells in driving autoimmune inflammation [51]. Th17 are myelin specific cells that traffic to the CNS where they secrete IL-17, which attracts other immune and myeloid cells in the CNS, perpetuating the inflammatory cascade [52].

The genes responsible for cytokine generation are highly influenced by the presence of single nucleotide polymorphisms (SNP) in main regions such as regulatory sequences or in promoter regions, contributing to disease susceptibility and evolution.

To our knowledge, this is the first study performed in the Romanian population which tried to evaluate the association between three SNPs, IL-27-*T4730C*, IL-27-*A964G* and IL-23-*R381Q*, and susceptibility to multiple sclerosis.

In this study, we investigated the influence of the genetic variability of IL-27 and IL-23R genes on MS. A significant difference in the frequency of genotypes of the studied IL-27 SNP and IL-23R SNP between patients with MS and controls was detected.

Over the years, studies have tried to offer a model of prevention in MS, selectively targeting IL-23 [53]. In this respect, studies started to evaluate SNP in IL-23R, showing that perturbations in IL-23 signaling pathways lead to pathophysiological changes which may be the ground for the development of autoimmune diseases such as MS, IBD, psoriasis, RA and AS [54,55].

According to the National Human Genome Research Institute catalogue of GWAS, the IL-23R gene is recognized for its involvement in developing autoimmune and inflammatory disorders [56]. Considering the crucial role of the IL-23 gene in generating Th17 cells, we selected one single nucleotide polymorphism to examine if the IL-23 gene polymorphism *G1142A* (rs11209026, R381Q) can be linked to MS susceptibility.

Hazlet et al. and Yu et al. evaluated the functional outcomes of *R381Q* (*G1142A*-rs11209026) polymorphism in RA, observing an altered function of IL-23, which determines a lowered production of Th17 cells and IL-17 upon IL-23 stimulation, suggesting a protective role for rs11209026 in some autoimmune diseases [57].

These results were also reported in a study conducted by Sarin et al. in inflammatory diseases (IBD and psoriasis), in which they concluded that IL-23-*R381Q* variant may confer protection against autoimmunity and excessive inflammation [58]. Moreover, Pidasheva et al., Di Meglio et al., Nossent et al., confirmed the protective role of the IL-23-*R381Q* for AS, psoriasis and Crohn disease (CD) [24,59,60].

Other studies such as those performed on patients with systemic sclerosis, MS, SLE and Sjogren’s syndrome and AS did not find a protective role for IL-23- *R381Q* polymorphism [55,61,62,63,64].

In our study, we obtained similar results to those obtained by Pidasheva et al., Di Meglio et al. and J.C Nossent et al. [24,59,60], confirming the association of IL-23- *R381Q* polymorphism with MS under the codominant model after adjustment for age, gender and smoking factors as covariates, as well as after correcting for multiple testing using the FDR method. The presence of the IL-23-*R381Q* heterozygous genotype can be protective for MS development. For the IL-23-*R381Q* polymorphism, under the codominant model, the frequency of the heterozygous genotypes was lower in MS patients as compared to healthy subjects. The heterozygous genotype decreased the odds for MS by 0.26-fold, suggesting that this polymorphism confers protection for MS.

The mechanism involved in the protective effect conferred by IL-23-*R381Q* variant could be due to reduced STAT3 phosphorylation and an altered conjunction between JAK2 proteins and the cytoplasmic end of the receptor for IL-23 [24]. Raymond et al. suggested another protective mechanism of IL-23-*R381Q,* in which this genetic variant disrupts a binding site for the splice enhancer protein SF2, lowering the activity of this protein in allele A carriers and elevating the expression of IL23RΔ9 splice form [21]. A truncated form of the protein is produced, which lowered IL-23 expression, decreasing the host’s ability to produce a TH17 phenotype upon IL-23 stimulation [21].

The substitution of Arg to Gln in codon 380 (*R381Q* variant) in autoimmune diseases causing different effects in different inflammatory conditions could be secondary to the multifactorial etiology of each disease, to the interactions between genetic and environmental factors that can influence the role of the IL-23R polymorphism in the pathogenetic process [65,66].

IL-27, the other member of the IL-12 family, is composed of IL-27p28 and the Epstein–Barr-induced gene 3 product (EBI3) [67].

The major source of IL-27 production is represented by antigen presenting cells (APC) activated by inflammatory mediators or microbial agents [68]. IL-27 presents antagonistic roles in inflammation. The proinflammatory role of IL-27 is mediated through CD4+ and CD8+ T cell activation. On the other hand, through IL-10 production, regulatory T (Treg) cell proliferation and programmed cell death ligand 1 (PD-L1) upregulation, IL-27 prevents the differentiation of Th17 cells and limits the inflammatory processes [69,70].

Ghavami et al. [39] evaluated one genetic polymorphism located in the IL-27 gene, *A964G*, in patients with acute lymphoblastic leukemia (ALL) and demonstrated a higher risk to develop ALL for patients presenting the AG heterozygous genotype and G allele, a greater relapse rate and a worse therapeutic response for these patients. Similar results were obtained by Pu et al. in patients with renal cancer and in patients with colorectal cancer, Moazeni-Roodi et al. in a meta-analysis including patients with gastrointestinal cancer, colorectal cancer and breast cancer, Jahantigh et al. in preeclamptic women [35,71,72,73].

Other genetic studies revealed that IL-27-*A964G* polymorphism influences messenger ribonucleic acid (mRNA) expression, predisposing to different inflammation-related diseases [74,75,76]. Junbing et al., Chae et al., Li et al. and H et al. demonstrated an association between sepsis, asthma, CD and Grave’s disease and the presence of the IL-27-*A964G* polymorphism [40,77,78]. Zhao et al. did not find any association between the pathophysiology of immune thrombocytopenic purpura (ITP) and the *A964G* polymorphism in the Chinese population [79].

In MS, the IL-27-*A964G* polymorphism has never been studied before. In our study, the IL-27-*A964G* gene polymorphism was significantly associated with increased odds of MS under codominant and dominant genetic models after adjustment for age, gender and smoking factors as covariates, but not in recessive and overdominant genetic models. After correcting for multiple testing, using the FDR method, these associations remained significant just under dominant genetic models. For IL-27-*A964G* polymorphism, only under the dominant model, the frequency of the AG + GG genotype was significantly higher in patients with MS compared to controls. Under the codominant model, the AG and GG genotypes increase the odds for MS by 1.77-fold and 2.44-fold respectively, but the results were not statistically significant after correcting for multiple testing using the FDR method. Under the dominant model, the AG + GG genotype of IL-27-*A964G* polymorphism increases the odds for MS by 1.93- fold. The results suggest that people carrying the AG + GG genotype are more likely to develop MS than people carrying other genotypes. The results were in agreement with those obtained by Paradowska-Gorycka et al. in RA patients, Chae et al. and Shen et al. in patients with asthma and allergic rhinitis [40,75,80].

Because the genetic polymorphism is located in the promoter of the IL-27p28 gene, it is suggested that it might affect gene splicing, transcription factor binding, or the non-coding RNA sequence, influencing the expression of certain proteins [80].

Different studies evaluated a possible correlation between IL-27-*T4730C* genetic variant and RA, insulin resistance, preeclampsia (PE) and HIV infection, but no associations were found between the genetic polymorphism, the expression of cytokines and these diseases [35,81,82,83].

Associations with malignancies were found for the IL-27-*T4730C* polymorphism, which was linked to the susceptibility of developing colorectal, hepatocellular and esophageal neoplasms, but it was found protective for UC [44,48].

He and colleagues found a strong correlation between the IL-27-*A964G* variant and GD, but no correlation was found between autoimmune thyroid diseases and the IL-27-*T4730C* variant [84]. We obtained preliminary results in a pilot study in which we analyzed the possible role of the IL-27-*T4730C* polymorphism in Multiple Sclerosis [85].

In the present study, regarding the IL-27-*T4730C* polymorphism, under the codominant model, the frequency of the TC and CC genotypes was significantly higher in patients with MS compared to controls. Under the dominant and overdominant models, the frequency of the TC + CC and CC genotypes was significantly higher in patients with MS compared to controls. The IL-27-*T4730C* gene polymorphism was significantly associated with increased odds of MS under the codominant, dominant, recessive and overdominant genetic models after adjustment for age, gender and smoking factors as covariates. After correcting for multiple testing using the FDR method, these associations remained significant under the codominant, dominant and overdominant genetic models except for the recessive model. Under the codominant model, carriers of the TC and CC genotype had 4.16-fold and 3.87-fold, respectively, increased odds to develop MS. The same results were obtained under the dominant and overdominant models, which showed that carriers of the TC + CC and TT + CC genotypes had 4.09-fold and 3.64-fold increased odds to develop MS.

We also tried to evaluate the interaction of SNPs as biomarkers for MS. However, our results showed that the odds for MS in association with one polymorphism is not influenced by the other investigated polymorphism. We found no studies that investigated this association.

Few studies have been considered a relation between haplotypes and the development of human disorders.

Li et al., Chae et al. and Huang et al. investigated the haplotypes of IL-27-*A964G*, IL-27-*T2905G*, and IL-27-*T4730C* and found an association with the susceptibility to IBD, asthma, and chronic obstructive pulmonary disease (COPD) [40,77,86].

In the present study, we evaluated the haplotype frequencies of these two IL-27 SNPs in both multiple sclerosis patients and healthy controls.

Our results showed a LD association between the IL = 27-*A964G* and IL-27-*T4730C* polymorphisms, D′ = 0.64 and r2 = 0.383.

An association analysis of these haplotypes with odds of multiple sclerosis revealed that only GC (IL-27-*A964G,* IL-27-*T4730C*) was significantly associated with MS risk in the additive model with adjustment for age group (≤40 years vs. >40 years), sex and smoking. The GC haplotype was positively correlated with MS; this haplotype might be associated with susceptibility to MS in Romanian populations. Patients with this haplotype are more prone to multiple sclerosis than those with other haplotypes. GT and AC haplotype frequencies did not differ significantly between the groups. The results are in agreement with those obtained by Paradowska-Gorycka et al., which found from four possible haplotypes of the IL-27-*A964G* and IL-27-*T4730C* genes (A-T, G-T, A-C, G-C), a higher frequency of the GC haplotype (*p* = 0.001062) in patients with RA than in controls [75].

In the present study, we investigated the association between the three SNPs and the demographic and clinical characteristics of MS patients. Our results suggested that even though higher frequencies were found for smoking and alcohol consumption in patients with MS carrying the IL-27-*T4730C,* IL-27-*A964G*, IL-23-*R381Q* polymorphisms compared to non-carriers, the results were not statistically significant. Regarding other parameters, no association with IL-27-*T4730C*, IL-27-*A964G*, IL-23- *R381Q* polymorphisms was found. There was a study which reported an association between these polymorphisms and clinical parameters, but the study was performed in patients with rheumatoid arthritis [75].

Although this report is the first study conducted in the Romanian population, our study has some limitations, which should be underlined. First, the design of our study was aimed at MS patients in only one population sample. Secondly, only three SNPs (IL-27-*T4730C*, IL-27-*A964G*, IL-23-*R381Q)* were investigated in our study. Thirdly, the results might be undervalued because of the limitation of the sample size. Therefore, additional multi-center and experimental studies in larger sample sizes should be assessed for better understanding the effects of gene polymorphisms on MS and the ethnicity-based genetic diversities.

## 5. Conclusions

Results of the current study highlighted a significant relationship of functional IL-27-*T4730C* and IL-27-*A964G* polymorphisms with increased risk of MS. Moreover, the haplotype-based analysis proposed the GC haplotype (IL-27-*A964G*, IL-27-*T4730C*) as a significant risk haplotype for MS. Our data showed the protective role of the IL-23-*R381Q* A allele against MS in studied population and future studies may extend the observation towards a protective clinical phenotype with less severe clinical disease, especially in heterozygous patients.

## Figures and Tables

**Table 1:** PCR and RFLP conditions for IL-27 and IL-23 polymorphism identification.

Genetic Variations	Primers	T_anneal_	PCR Fragments	Restriction Enzymes	Fragments
IL-27-*T4730C* (Leu119Pro) *(rs 181208)*	FW:5′- GCTTCAGCCCTTCCATGCCC-3′ RW:5′-TCTACCTGGAAGCGGAGGTGCC-3′	67 °C	132 bp	*FuaI*	*T4730* allele: 132 bp *C4730* allele: 112, 20 bp
IL-27-*A964G* *(rs 153109)*	FW:5′-GGCTGTGCTGGAAGGGAGAC-3′ RW: 5-ATATCTGGGACCAGGGTTAGG-3′	62 °C	468 bp	*XhoI*	*A964* allele: 468 bp *G964* allele: 347, 121 bp
IL-23-*G1142A* (R381Q) (rs 11209026)	FW:5′-CTTTTCTGGCAGGGTCATTTTG-3′ RW:5′-AAGTTGTTTCCTGGGGTAGTTGTG-3′	60 °C	508 bp	*Hpy188i*	*G1142* allele: 288, 103, 82, 35 bp *A1142* allele: 323, 35 bp

**Table 2:** Distribution of demographic, clinical and lifestyle characteristics in MS patients and controls.

Category Data	Variables		Controls (*n*_1_ = 95)	MS Patients (*n*_2_ = 157)	*p*-Value
Demographic	Age, years ^(a)^		27 (25,32)	42 (31,50)	<0.001 *
	Sex ^(b)^				
	Male		34 (35.8)	51 (32.5)	0.591
	Female		61 (64.2)	106 (67.5)	
	Residence ^(b)^				
	Urban area		95 (100.0)	122 (77.7)	<0.001 *
	Rural area		0 (0.0)	35 (22.3)	
Lifestyle	Smoking ^(b)^		20 (21.1)	52 (33.1)	0.040 *
	Alcohol consumption ^(b)^		59 (62.1)	35 (22.4)	<0.001 *
Clinical	Age at diagnosis, years ^(c)^		NA	30 (24, 40)	
	Duration of the disease ^(b)^				
	<5 years		NA	62 (39.5)	
	5–10 years		NA	27 (17.2)	
	>5 years		NA	68 (43.3)	
	Form of MS ^(b)^				
	CIS		NA	26 (16.6)	
	RR		NA	109 (69.4)	
	SP		NA	22 (14.0)	
	EDSS ^(c)^		NA	2.0 (1.0, 3.0)	
	Disease treatment ^(a)^		NA	109 (69.4)	

MS–multiple sclerosis; Data are shown as ^(a)^ mean ± standard deviation or ^(b)^ absolute frequencies (percentages estimated from the size of the group or ^(c)^ median [Q1, Q3]); values denoted by * indicate a significant result (*p*-value < 0.05); NA = not applicable; CIS—clinically isolated syndrome; RR—relapsing remitting; SP—secondary progressive; EDSS—expanded disability status scale.

**Table 3:** Allelic and genotypic distributions of SNPs of IL-23 and IL-27 gene polymorphisms in MS patients and controls.

SNPs	Genetic Model	Genotypes	Controls ^(a)^ (*n*_1_ = 95)	MS Patients ^(a)^ (*n*_2_ = 157)	OR [95% CI]	*p*-Value Unadjusted	OR [95% CI] ^(b)^	*p*-Value Adjusted ^(b)^	Q-Value ^(c)^
IL-27-*T4730C*	Codominant	TT	76 (80.0)	86 (54.8)	1.00	0.0002 *	1.00	0.00004 *	0.00018 *
		TC	15 (15.8)	52 (33.1)	3.06 [1.60, 5.88]		4.16 [2.04, 8.48]		
		CC	4 (4.2)	19 (12.1)	4.20 [1.37, 12.88]		3.87 [1.17, 12.81]		
	Dominant	TT	76 (80.0)	86 (54.8)	1.00	0.00003 *	1.00	0.000007 *	0.000063 *
		TC + CC	19 (20.0)	71 (45.2)	3.30 [1.83, 5.97]		4.09 [2.14, 7.83]		
	Recessive	TT + TC	91 (95.8)	138 (87.9)	1.00	0.026 *	1.00	0.083	0.1067
		CC	4 (4.2)	19 (12.1)	3.13 [1.03, 9.51]		2.64 [0.82, 8.51]		
	Overdominant	TT + CC	80 (84.2)	105 (66.9)	1.00	0.00019 *	1.00	0.00015 *	0.00045 *
		TC	15 (15.8)	52(33.1)	3.13 [1.03, 9.51]		3.64 [1.80, 7.36]		
IL-27-*A964G*	Codominant	AA	37 (38.9)	39 (24.8)	1.00	0.0428 *	1.00	0.0727	0.1067
		AG	44 (46.3)	82 (52.2)	1.77 [0.99, 3.16]		1.77 [0.94, 3.33]		
		GG	14 (14.7)	36 (22.9)	2.44 [1.14, 5.24]		2.43 [1.06, 5.55]		
	Dominant	AA	37 (38.9)	39 (24.8)	1.00	0.0189 *	1.00	0.0324 *	0.05832 ^#^
		AG + GG	58 (61.1)	118 (75.2)	1.93 [1.12, 3.34]		1.93 [1.05, 3.51]		
	Recessive	AA + AG	81 (85.3)	121 (77.1)	1.00	0.1082	1.00	0.1425	0.16031
		GG	14 (14.7)	36 (22.9)	1.72 [0.87, 3.39]		1.71 [0.82, 3.55]		
	Overdominant	AA + GG	51 (53.7)	75 (47.8)	1.00	0.3627	1.00	0.4075	0.4075
		AG	44 (46.3)	82(52.2)	1.27 [0.76, 2.11]		1.26 [0.73, 2.20]		
IL-23-*R381Q*	Codominant	GG	85 (89.5)	153 (97.5)	1.00	0.0084 *	1.00	0.0276 *	0.05832 ^#^
		AG	10 (10.5)	4 (2.5)	0.22 [0.07, 0.73]		0.26 [0.08, 0.92]		
		AA	0 (0.0)	0 (0.0)	─		─		

MS- multiple sclerosis; ^(a)^ Absolute number and percentage (estimated from the group size) of individuals; ^(b)^ ORs and *p*-values adjusted for age group (≤40 years vs. >40 years), sex and smoking; adjusted p-values denoted by * indicate statistically significant results (*p*-value < 0.05); ^#^ significant corrected *p*-values for multiple comparisons (Q-value < 0.10) ^(c)^ False Discovery Rate (FDR) adjustment for multiple testing.

**Table 4:** Association analysis of SNP-SNP interactions with odds of MS.

Gene Polymorphisms	Genetic Model	IL-27-*A964G*	IL-27-*T4730C*	IL-23-*R381Q*
IL-27-*A964G*	Codominant	0.0423/0.069	0.294/0.195	0.106/0.267
	Dominant	0.018/0.029	0.063/0.093	0.089/0.171
	Recessive	0.115/0.155	0.584/0.575	NA
IL-27-*T4730C*	Codominant		0.0002/<0.001	0.536/0.269
	Dominant		<0.0001/<0.0001	0.511/0.282
	Recessive		0.035/0.099	NA
IL-23-*R381Q*	Codominant			0.007/0.024
	Dominant			0.007/0.024
	Recessive			NA

Note. The *p*-values of epistatic pairwise interactions were indicated in the upper part of the matrix and were obtained using the log likelihood ratio test (LRT). The *p*-values from the diagonal of the matrix represented the unadjusted effect of all investigated SNPs on MS odds; NA = not applicable (missing AA genotype); after the slash symbol there are the *p* values adjusted for age group (≤40 years vs. >40 years), sex and smoking.

**Table 5:** Haplotype analysis for association of IL-27 gene polymorphisms (*A964G* and *T4730C*) with MS odds.

Haplotypes	Total Relative Frequency	Haplotype Frequencies in the Control Group	Haplotype Frequencies in MS Patients	Hap-Score ^(a)^	*p*-Value ^(b)^	Permutation *p*-Value ^(c)^	OR ^(d)^ [95% CI]	OR ^(e)^ [95% CI]
A—T	0.5074	0.5809	0.4631	−2.715	0.0066 *	0.0060 *	1.00 [Reference]	1.00 [Reference]
G—T	0.2684	0.2980	0.2503	−0.998	0.3174	0.3187	1.14 [0.73, 1.76]	1.10 [0.71, 1.71]
A—C	0.0442	0.0401	0.0465	0.709	0.4782	0.4748	1.32 [0.47, 3.73]	1.37 [0.48, 3.87]
G—C	0.1799	0.0809	0.2401	4.02	0.00006 *	0.00005 *	3.20 [1.74, 5.88]	3.18 [1.74, 5.81]

MS–multiple sclerosis; Global test of association for the additive haplotype model Global- Statistics = 16.81, df = 3, overall *p*-value = 0.00077. Note. Alleles in the haplotype were described in order of studied gene polymorphisms (IL-27-*A964G* and IL-27-*T4730C*); additive haplotype model adjusted for age group (≤40 years vs. >40 years), sex and smoking; haplotypes estimated from the two variants are ordered according to the Score test statistics; ^(a)^ haplotype frequencies inferred by haplo.stats; haplotype score for the haplotype; ^(b)^ empirical *p*-values of the corresponding Score test; ^(c)^ simulation *p*-value based on 20,000 bootstrap replicates; ^(d)^ effect sizes of each haplotype estimated from haplotype-based GLM regression without covariates; ^(e)^ effect sizes of each haplotype adjusted for age group (≤40 years vs. >40 years), sex and smoking; * *p*-values denoted by * indicated significant results (*p* < 0.05).

**Table 6:** Association between IL-27-*T4730C*, IL-27-*A964G*, IL-23-*R381Q* and clinical characteristics of multiple sclerosis.

Variables	IL-27-*A964G*			IL-27-*T4730C*			IL-23-*R381Q*		
	AA (*n*_1_ = 39)	AG/GG (*n*_2_ = 118)	*p*-Value	TT (*n*_1_ = 86)	TC/CC (*n*_2_ = 71)	*p*-Value	GG (*n*_1_ = 153)	AG (*n*_2_ = 4)	*p*-Value
Age, years ^(a)^	42.00 ± 12.58	40.97 ± 10.76	0.619	42.19 ± 11.88	40.06 ± 10.29	0.237	41.20 ± 10.98	42.00 ± 20.38	0.888
Age at diagnosis, years ^(b)^	30 (23.5, 39.5)	30 (26,40)	0.642	29.5 (24.25, 39.75)	30 (25,40)	0.951	30 (25,40)	24.50 (22.25, 30)	0.308
Sex (male) ^(c)^	15 (38.46)	36 (30.51)	0.358	28 (32.56)	23 (32.39)	0.983	50 (32.68)	1 (25.00)	1.000
Residence (urban) ^(c)^	28 (71.79)	94 (79.66)	0.306	68 (79.07)	54 (76.06)	0.652	118 (77.12)	4 (100.00)	0.576
Smoking ^(c)^	11 (28.21)	41 (34.75)	0.452	28 (32.56)	24 (33.80)	0.869	50 (32.68)	2 (50.00)	0.600
Alcohol consumption ^(c)^	8 (20.51)	27 (23.08)	0.739	19 (22.35)	16 (22.54)	0.978	33 (21.71)	2 (50.00)	0.218
Duration of the disease ^(c)^			0.507	0.507		0.252			0.291
<5 years	13 (33.33)	49 (41.53)		32 (37.21)	30 (42.25)		61 (39.87)	1 (25.00)	
5–10 years	6 (15.38)	21 (17.80)		12 (13.95)	15 (21.13)		25 (16.34)	2 (50.00)	
>10 years	20 (51.28)	48 (40.68)		42 (48.84)	26 (36.62)		67 (43.79)	1 (25.00)	
Personal history of autoimmune diseases ^(c)^	1 (2.56)	4 (3.39)	1.000	3 (3.49)	4 (2.82)	1.000	4 (2.61)	1 (25.00)	0.123
Form of MS ^(c)^			0.091			0.259			0.772
CIS	8 (20.51)	18 (15.25)		17 (19.77)	9 (12.68)		25 (16.34)	1 (25.00)	
RR	22 (56.41)	87 (73.73)		55 (63.95)	54 (76.06)		106 (69.28)	3 (75.00)	
SP	9 (23.08)	13 (11.02)		14 (16.28)	8 (11.27)		22 (14.38)	0 (0.00)	
EDSS Score at admission ^(b)^	2.0 (1.0, 3.5)	2.0 (1.0, 3.0)	0.399	2.0 (1.0, 3.0)	2.0 (1.0, 3.5)	0.793	2.0 (1.0, 3.5)	1.5 (1.4, 1.8)	0.577

Data are shown as ^(a)^ mean ± standard deviation, ^(b)^ median [Q1, Q3] or ^(c)^ absolute frequencies (% percentages estimated from the size of the group); *p*-values were obtained from Student’s *t*-test for independent samples, Mann-Whitney U test, Chi-square or Fisher’s exact tests. CIS—clinically isolated syndrome; RR—relapsing remitting; SP—secondary progressive; EDSS—expanded disability status scale.

## Data Availability

The raw data involved in this study can be obtained upon reasonable request addressed to Lucia M. Procopciuc (luciamariaprocopciuc@yahoo.com) and Ioana S. Barac (siminabarac@gmail.com).
